# Differential regulation of amphetamine-induced serotonergic and dopaminergic efflux by syntaxin 1A

**DOI:** 10.1186/1471-2210-11-S2-A38

**Published:** 2011-09-05

**Authors:** Therese Montgomery, Sonja Sucic, Thomas Steinkellner, Michael Freissmuth, Harald Sitte

**Affiliations:** 1 Institute of Pharmacology, Center of Physiology and Pharmacology, Medical University of Vienna, 1090 Vienna, Austria

## Background

The plasma membrane serotonin transporter (SERT) is a key regulator of synaptic serontonergic neurotransmission and is a major target of both antidepressents and psychostimulant drugs of abuse. The pre-synaptic soluble *N*-ethylmaleimide-sensitive factor attachment protein receptor (SNARE) protein syntaxin 1A has been reported to modulate the intrinsic activity of multiple monoamine neurotransmitter transporters both *in vitro* and *ex vivo*. However, in contrast to the dopamine transporter (DAT) little is know of its effect on SERT-dependent amphetamine-mediated efflux in neuronal cells. Thus, the purpose of this study was to examine the specific effects of syntaxin 1A on both SERT function and regulation by common drugs of abuse.

## Methods

Murine catecholaminergic cells (CAD cells) were transiently transfected with either DAT or SERT in the presence and absence of syntaxin 1A. Transporter function was assessed by [^3^H]MPP+ and [^3^H]5-HT uptake, respectively. The cells were pre-loaded with [^3^H]MPP+ and superfused with amphetamines in order to determine the effect of syntaxin 1A co-expression on amphetamine-mediated transporter-dependent efflux. Mutagenesis was performed using the QuikChange II site-directed mutagensis kit from Stratagene. Transporter and syntaxin 1A co-localisation was confirmed using confocal microscopy.

## Results

The co-expression of SERT and syntaxin 1A led to a significant reduction in the V_max_, but not the K_m_, for [^3^H]5-HT uptake. Similarly, syntaxin 1A co-expression greatly reduced parachloroamphetamine-induced SERT-dependent efflux. Neither the pharmacological inhibition of CaMKII, nor the mutation of a CaMKII-binding motif in the N-terminal tail of SERT had any effect on the down-regulation of SERT activity by syntaxin 1A in neuronal cell lines. In contrast to SERT, the co-expression of syntaxin 1A and DAT had no effect on [^3^H]MPP+ uptake via DAT. Moreover, D-amphetamine-induced efflux via DAT was increased by the co-expression of syntaxin 1A.

## Conclusions

In contrast to DAT, syntaxin 1A is a negative regulator of amphetamine-induced SERT-mediated efflux, an effect which occurs independently of CaMKII activation. The significance of this differential regulation is currently being investigated using endogenous expression systems.

